# Developmental Phenotypic and Transcriptomic Effects of Exposure to Nanomolar Levels of 4-Nonylphenol, Triclosan, and Triclocarban in Zebrafish (*Danio rerio*)

**DOI:** 10.3390/toxics10020053

**Published:** 2022-01-24

**Authors:** Jessica Phillips, Alex S. Haimbaugh, Camille Akemann, Jeremiah N. Shields, Chia-Chen Wu, Danielle N. Meyer, Bridget B. Baker, Zoha Siddiqua, David K. Pitts, Tracie R. Baker

**Affiliations:** 1 Institute of Environmental Health Sciences, Wayne State University, Detroit, MI 48202, USA; jessica.montrief@wayne.edu (J.P.); alexhaim@wayne.edu (A.S.H.); cakemann14@gmail.com (C.A.); jshields@wayne.edu (J.N.S.); chiachenwu@ufl.edu (C.-C.W.); danielle.meyer@ufl.edu (D.N.M.); bridgetbaker@ufl.edu (B.B.B.); 2 Department of Pharmacology, Wayne State University, Detroit, MI 28201, USA; 3 Department of Environmental and Global Health, University of Florida, Gainesville, FL 32610, USA; 4 Department of Wildlife Ecology and Conservation, University of Florida, Gainesville, FL 32610, USA; 5 Department of Pharmaceutical Sciences, College of Pharmacy and Health Sciences, Wayne State University, Detroit, MI 48202, USA; zoha.siddiqua@wayne.edu (Z.S.); pitts@wayne.edu (D.K.P.)

**Keywords:** triclosan, triclocarbon, detergents, 4-nonylphenol, *Danio rerio*, zebrafish, environmental toxicity, development, aquatic environment, ground water chemicals

## Abstract

Triclosan, triclocarban and 4-nonylphenol are all chemicals of emerging concern found in a wide variety of consumer products that have exhibited a wide range of endocrine-disrupting effects and are present in increasing amounts in groundwater worldwide. Results of the present study indicate that exposure to these chemicals at critical developmental periods, whether long-term or short-term in duration, leads to significant mortality, morphologic, behavioral and transcriptomic effects in zebrafish (*Danio rerio)*. These effects range from total mortality with either long- or short-term exposure at 100 and 1000 nM of triclosan, to abnormalities in uninflated swim bladder seen with long-term exposure to triclocarban and short-term exposure to 4-nonylphenol, and cardiac edema seen with short-term 4-nonylphenol exposure. Additionally, a significant number of genes involved in neurological and cardiovascular development were differentially expressed after the exposures, as well as lipid metabolism genes and metabolic pathways after exposure to each chemical. Such changes in behavior, gene expression, and pathway abnormalities caused by these three known endocrine disruptors have the potential to impact not only the local ecosystem, but human health as well.

## 1. Introduction

Since the Industrial Revolution, humans have created and used chemicals as a part of technological advancements necessary to meet the demands of the world’s exponentially growing population. Unfortunately, most chemicals are put into use before the full extent of their health effects on humans and wildlife is known. In the past, chemicals have been released into the environment via dumping in lakes, rivers, or into the ground before regulations like the Resource Conservation and Recovery Act (RCRA) were put in place for chemical waste disposal. Despite regulations like these, chemicals are still used and released into the environment, and many are resistant to degradation with the potential to cause adverse effects on local ecosystems that extend to wildlife and humans. For instance, the U.S. Food and Drug Administration released a Final Rule in 2016 regarding the antimicrobial triclosan, that concluded human and ecosystem health is not sufficiently protected from the adverse impacts of antimicrobial and antiseptic chemicals by existing regulatory practices [1].

As research increases our knowledge of these environmental contaminants and their properties, we are discovering that many are endocrine disrupting chemicals (EDCs). According to the National Institute of Environmental Health Sciences (NIEHS), EDCs are “chemicals that may interfere with the body’s endocrine system and produce adverse developmental, reproductive, neurological, and immune effects in both humans and wildlife” [1]. Sources of EDCs are wide ranging and include industrial processes, personal care products, pharmaceuticals, pesticides, and more. Triclosan and triclocarban are antimicrobial agents now banned in soaps in the United States due to evidence that they do not prevent disease or improve health but are toxic and carcinogenic, mainly via endocrine disruption [2]. Nonetheless, they are still found in personal care products such as lotions, deodorants, and toothpaste, such as Colgate, which contains 10 mM of triclosan, then enter the environment mainly through wastewater effluent [3,4] where they accumulate due to their resistance to biodegradation, with an approximate average of 200 ng/L found for both in U.S. surface waters [5]. Triclosan and triclocarban also have the ability to cross the placental barrier [6]. Pregnant rats exposed to triclosan had dramatically decreased serum levels of estradiol, progesterone, and prolactin, as well as differential expression of genes responsible for hormone biosynthesis targeting the placenta [7]. Studies have shown that triclosan exposure may be linked to decreased oocyte implantation in women struggling with fertility [8] and may have placental endocrine effects [9]. In fact, human fecundity decreases when triclosan concentrations exceed 75 ng/mL in urine [10]. Human studies also showed disruption of thyroid hormones, specifically triclosan has a positive association with circulating levels of triiodothyronine [11]. Similarly, triclocarban exposure disrupts thyroid hormones in human cell lines and frogs [12], increases androgenic activity in human cell lines [13], reduces female plasma vitellogenin and estradiol in female fathead minnows, resulting in approximately half the cumulative egg production compared to unexposed fish, and decreases testosterone while increasing estradiol in male fathead minnows at environmentally relevant levels [14].

4-nonylphenol is another known EDC that can be found in a wide range of products including fungicides, food packaging, toys, clothes, jewelry, and cosmetics [15]. 4-nonylphenol causes health effects such as liver toxicity and steatosis in male rats [16] as well as induces hormone disruption by inhibiting progesterone and androstenedione [17]. Additionally, exposure to 4-nonylphenol during critical developmental periods such as puberty leads to decreased testosterone production and spermatogenesis, as well as increased morphological sperm abnormalities in rats [18]. Humans are not only exposed to these chemicals directly in consumer products, but also via the environment where they are found ubiquitously in surface water, drinking water, wastewater effluent, soil, and wildlife [19,20,21,22,23,24].

Although these EDCs have been studied in many different organisms, little is known about the health consequences of exposure to environmentally relevant levels at critical developmental windows, and the acute and later life effects of these early life exposures. The purpose of this study is to evaluate the health risks of early life exposure to EDCs commonly found at low but persistent levels in the environment. Thus, we exposed zebrafish (*Danio rerio)* larvae to triclosan, triclocarban, or 4-nonylphenol during early development, and examined its effects on morphology, behavior, and gene expression. Zebrafish are an NIH-approved human model because their genome shares 70% homology with the human genome. Organogenesis takes place within the first 42 h post-fertilization, whereas hatching occurs around 48 h post-fertilization, at which point the larval stage begins [25]. Zebrafish can also produce hundreds of eggs per week, making them an excellent model for high-throughput screening for multiple chemicals and endpoints. Additionally, since zebrafish are swimming freely by 3 days post-fertilization (dpf), we can measure neurobehavioral endpoints in early life. We found that exposure to triclocarban, triclosan, and 4-nonylphenol during these two critical development periods, embryogenesis and the early larval stage, can have detrimental morphological, behavioral, and transcriptomic effects, providing insight into timing of exposure, targets, and mechanisms of EDC toxicity.

## 2. Material and Methods

### 2.1. Animal Husbandry

The adult zebrafish (wild-type AB strain) used to spawn the larval fish for the experiments were maintained on a 14:10 light/dark cycle in reverse osmosis (RO) water buffered with salt (Instant Ocean© Spectrum Brands, Blacksburg, VA, USA) with temperature maintained at 27 °C–30 °C in a recirculating system (Aquaneering, San Diego, CA, USA). Adult fish were monitored and fed fish flakes twice daily (Aquatox Fish Diet, Zeigler Bros Inc., Gardners, PA, USA), with feeding supplemented by brine shrimp. Adult zebrafish were bred in spawning tanks with a sex ratio of 1 male to 2 females, and embryos were collected 4 h post-fertilization (hpf). The embryos were cleaned with bleach 58 ppm for 5 min (Clorox Company, Oakland, CA, USA), rinsed with RO water and then egg water (600 mg/L salt in RO water), sorted into exposure groups for their respective chemicals, and incubated at 28 °C. Zebrafish use protocols were approved by the Institutional Animal Care and Use Committee at Wayne State University, according to the National Institutes of Health Guide to the Care and Use of Laboratory Animals (Protocol 16-03-054; approved 4 August 2016).

### 2.2. Chemical Exposures

4-nonylphenol (CAS# 104-40-5, Sigma Aldrich, USA), triclocarban (CAS# 101-20-2, U.S. Pharmacopeia, Rockville, MD, USA), and triclosan (CAS# 3380-34-5, U.S. Pharmacopeia, Rockville, MD, USA) were used to prepare stock solutions. The 4-nonylphenol solutions were prepared in fish water (60 mg/L salt in RO water) at concentrations of 0.1, 1, 10, 100 and 1000 nM. The triclosan solutions were prepared in acetone at concentrations of 0.1, 1, 10, 100 and 1000 nM. The triclocarbon solutions were prepared in acetone at concentrations of 0.01, 0.1, 1, 10 and 100 nM. Control fish for the triclosan and triclocarban exposures were placed in vehicle (0.01% acetone (*v*/*v*) in RO water). The chemical solutions for the exposures were prepared daily from aliquots of the stock solutions. Larval chemical exposures were performed in 6-well plates, with embryos at a density of 30 per well and per exposure concentration, at one of two different time periods: either 120 h from 4 hpf to 5 days post-fertilization (dpf; “long-term exposure”); or 24 h at 4–5 dpf (“short-term exposure”). This was replicated 5 times, for a total of 150 larval fish exposed for each chemical, concentration, and duration of exposure. Approximately 90% of the chemical solution was removed from each well daily and replenished with freshly prepared chemical solution. After the exposure period, larval fish were rinsed 3 times with egg water to end the chemical exposure.

### 2.3. Abnormality Screening

Zebrafish embryos were screened at 24, 48, 72, 96, and 120 hpf for mortality and morphological abnormalities under stereomicroscope (M165C, Leica Microsystem, Wetzlar, Germany). The mortality endpoints assessed were coagulation of embryo and lack of heartbeat. The abnormality endpoints assessed were number of unhatched embryos compared to hatched embryos, skeletal deformities, improperly inflated swim bladder, yolk sac edema, cardiac edema, and total abnormalities. Embryos were screened using 6.7× magnification, with detailed evaluation occurring at a magnification of 20×. Results were analyzed for each chemical using a Chi-Square test with significance set at *p* < 0.05, with pairwise comparison with Bonferroni corrections.

### 2.4. Behavioral Analysis

At 5 dpf, 24 larval zebrafish from each exposure group and control groups with inflated swim bladders and without any morphological abnormalities were tested with 1 larva per well in a 24-well plate with 2 mL of fish water per plate. Each plate was allowed to acclimate for at least an hour at 27 °C before being placed in to the DanioVision Chamber (Noldus Information Technology, Wageningen, The Netherlands) to undergo the behavioral assay. The behavioral assay consisted of 3 min light and dark alternating periods, with a total of four light-dark cycles (24 min in total) and took place between 14:00 and 22:00. The integration time was set to 6 s and raw data files were processed using custom R scripts [26]. The behavioral endpoints assessed were as follows: Behavioral testing was performed between 1400 and 2200 h using fish that had acclimated in visible light. The raw data was exported from EthoVisionXT14 into a spreadsheet to perform quality control. The data series were not normally distributed, as normality was tested via a Shapiro–Wilks test. Interquartile range (IQR) method was used to remove outliers from light cycles. Data series were excluded from the overall light series if two serial data points were larger than 75th percentile plus 1.5 of IQR after the 1:00 min mark. In the dark cycle, data series were excluded if two serial data points were smaller than the median of the light data series. Finally, data series with a mean ratio of light:dark series equal or larger than 0.9 were removed. The behavioral data were then analyzed using ANOVA and Tukey’s HSD tests. Significance was considered at *p*-value smaller than 0.05. The quality control and statistics were conducted using R (http://www.r-project.org accessed on: 13 July 2021).

### 2.5. Transcriptomics

At 5 dpf, five larval fish per chemical concentration were pooled per tube in RNALater™ (Thermo Fisher Scientific, Waltham, MA, USA) at 4 °C. RNALater™ was removed after 24 h and samples were kept at −80 °C until RNA extraction. RNA isolation was performed using the RNeasy Lipid Tissue Mini Kit (QIAGEN, Hilden, Germany) according to manufacturer recommendations. RNA purity was measured with Qubit^®^ 3.0 Fluorometer (Invitrogen, Waltham, MA, USA) and RNA was stored at −80 °C until Quantseq library preparation. Quantseq 3′ mRNA-seq libraries were prepared from isolated RNA using QuantSeq 3′ mRNA-Seq Library Prep Kit FWD for Illumina (Lexogen, Vienna, Austria). Samples were normalized to 40 ng/µL (total input of 200 ng in 5 µL) and amplified at 17 cycles. Libraries were quantified using a Qubit^®^ 2.0 Fluorometer and Qubit^®^ dsDNA Broad Range Assay Kit (Invitrogen, Carlsbad, CA, USA), and run on an Agilent TapeStation 2200 (Agilent Technologies, Santa Clara, CA, USA) for quality control. The samples were sequenced on a HiSeq 2500 (Illumina, San Diego, CA, USA) in rapid mode (single-end 50 bp reads). Reads were aligned to D. rerio (Build danRer10) using the BlueBee Genomics Platform (BlueBee, Rijswijk, The Netherlands). Differential gene expression between the control and exposure lineage zebrafish was evaluated using DEseq2 (available through GenePattern; Broad Institute, Cambridge, Massachusetts). Genes with significant changes in expression, as defined by absolute log2-fold change value ≥0.75 and adjusted *p*-value < 0.1 were uploaded into Ingenuity Pathway Analysis software (IPA; QIAGEN Bioinformatics, Redwood City, CA, USA) for analysis using RefSeq IDs as identifiers.

## 3. Results

### 3.1. Triclosan

#### 3.1.1. Larval Abnormalities and Mortality

**Figure 1:**
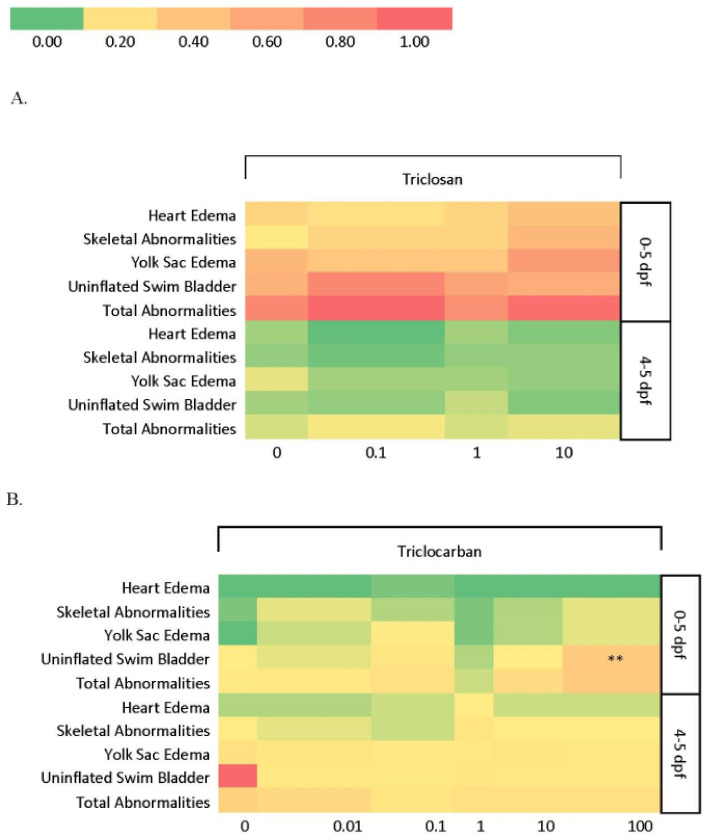

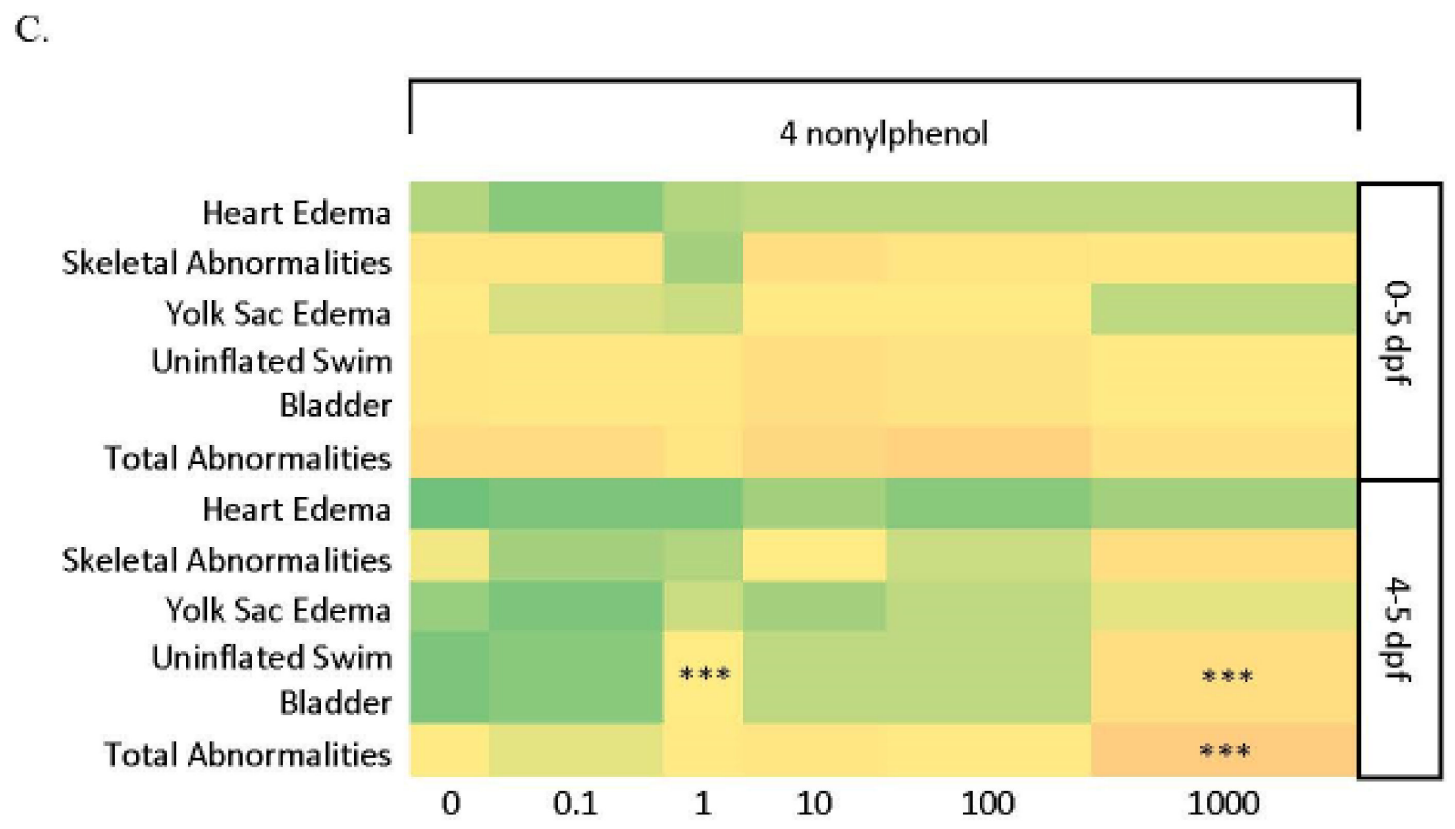
Heat map showing abnormality rate of zebrafish exposed to triclosan (**A**), triclocarban (**B**), or 4-nonylphenol (**C**) starting from 4 h post-fertilization to 5 days post-fertilization (0–5 dpf) or 4–5 dpf. ** indicates significant difference from control (*p* < 0.10), *** (*p* < 0.001).

No significant larval abnormalities were found at any concentration of triclosan following either the 24 or 120 h exposure (Figure 1A). The two highest triclosan concentrations (100 and 1000 nM) resulted in significant mortality following both the 24 and 120 h exposures, with all larval fish dying by 5 dpf in the 120 h exposure (*p* < 0.001). Because of the high mortality rate at these concentrations, abnormalities and behavior could not be evaluated. The percentage of unhatched eggs was significantly decreased in the 120 h exposure in the 10 nM concentration exposure group compared to control (*p* < 0.01).

#### 3.1.2. Behavior

**Figure 2:**
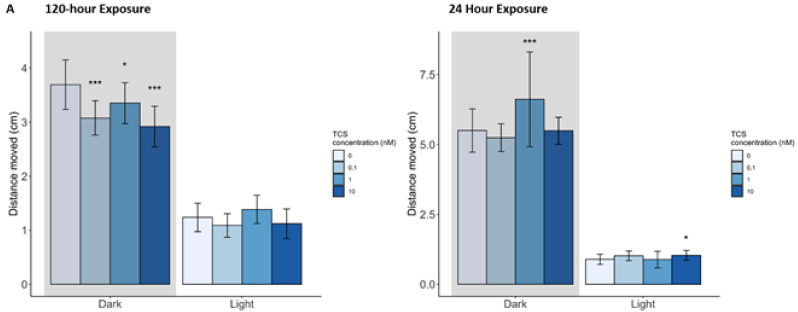

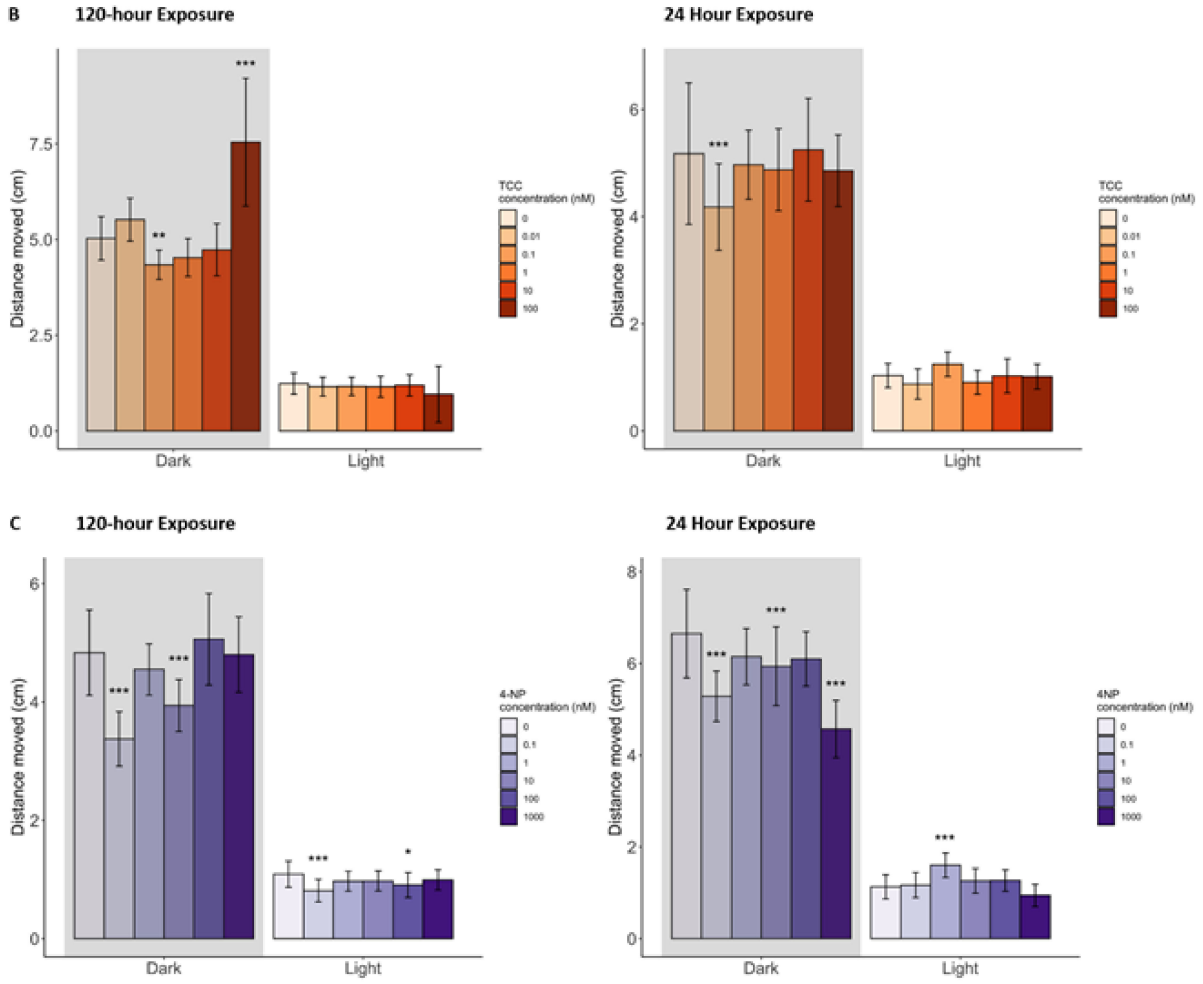
Average distance moved (cm) by larval zebrafish during light and dark cycles following (**A**) triclosan (TCS), (**B**) triclocarbon (TCC), or (**C**) 4-nonylphenol (4NP) exposure starting from 4 h post-fertilization to 5 days post-fertilization (dpf; 120 h exposure) or 4–5 dpf (24 h exposure): * indicates significant difference from control (*p* < 0.05), ** (*p* < 0.10), *** (*p* < 0.001); bars represent standard deviation.

The 120 h triclosan exposure resulted in significant behavioral changes in each concentration compared to controls with significant decreases in distance moved during the dark cycle following 0.1 and 10 nM exposures (*p* < 0.001), as well as 1 nM exposure (*p* < 0.05; Figure 2A). No significant difference in distance moved was observed during the light cycle for the 120 h exposure groups. In the 24 h exposure, however, movement was increased in both the dark cycle for the 1 nM exposure group (*p* < 0.001) and in the light cycle for the 10 nM exposure group (*p* < 0.05; Figure 2A).

#### 3.1.3. Gene Expression and Pathway Analysis

Table 1 shows the number and direction of change for differentially expressed genes (DEGs) following triclosan exposure. The 120 h exposure group resulted in 31 DEGs with absolute log2-fold changes ≥ 0.75 and adjusted *p*-values < 0.1, with 17 upregulated and 14 downregulated across all triclosan concentrations. For the 24 h exposure group, there were 45 DEGs across all concentrations with 35 upregulated and 10 downregulated (Table S1). The lowest triclosan concentration (0.1 nM) had the most DEGs regardless of exposure duration, with 19 and 22 DEGs following the 120 and 24 h exposures, respectively, 9 of which were commonly dysregulated by both exposure durations. The significant gene expression profiles were distinct for each exposure concentration following the 24 h exposure, except for hemoglobin, alpha embryonic 1.1 (*hbae1.3*) which was upregulated at 1 and 10 nM. The 120 h exposure was similar, with only one gene, cytochrome P450, family 2, subfamily K, polypeptide 18 (*cyp2k18*), differentially expressed after both 1 and 10 nM exposures. 

**Figure 3:**
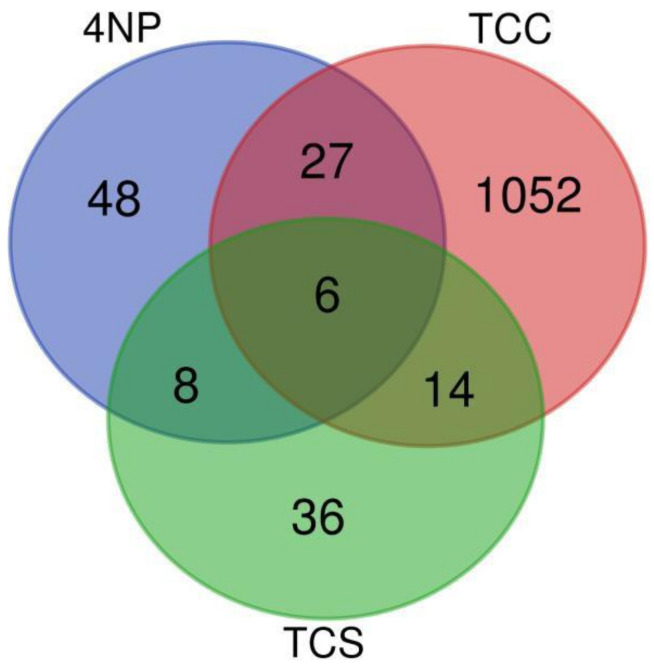
Venn diagram of differentially expressed genes after exposure to triclosan (TCS), triclocarban (TCC) or 4-nonylphenol (4NP) with all exposure concentrations and durations combined. The genes included in this diagram have an adjusted *p*-value < 0.1 and absolute log2-fold changes >0.75.

Triclosan and 4-nonylphenol shared 8 DEGs (Figure 3) in the following pathways: lipid metabolism, organ development, organ injury and abnormalities, and cancer. The SPINK1 pancreatic cancer pathway was the main pathway expressed following the 24 h triclosan exposure and 120 h 4-nonylphenol exposure, and includes genes such as carboxypeptidase A1 (pancreatic; *cpa1*), which was dysregulated at 1 nM triclosan, and 10 and 1000 nM 4-nonylphenol. For the 24 h duration triclosan exposure, the SP1NK pancreatic cancer pathway had 5 DEGs at 1 nM, while there was only 1 DEG at the 10 nM concentration. Additionally, the 120 h exposure groups for both triclosan and triclocarban shared 14 DEGs (Figure 3) in pathways involving: metabolic processes, including cholesterol biosynthesis, specifically following 1000 nM triclosan and 10 nM triclocarban; xenobiotic processes, specifically following 0.1 and 1 nM triclosan, as well as 1 and 10 nM triclocarban; organ development and morphology, particularly in the cardiovascular and neurological systems, specifically following 0.1, 1, and 1000 nM triclosan and 10 nM triclocarban. Table 2 shows the 14 genes commonly dysregulated by triclosan and triclocarban. There were six DEGs across all three chemicals, including genes related to: cardiovascular system development, such as F-box protein 32 (*fbxo32*) and hemoglobin, alpha embryonic 1.3 (*hbae1.3*); intracellular processes, such as mitochondrial trna (*mt-trna*), si:ch211-153b23.4 (*si:ch211-153b23.4*), and heterogeneous nuclear ribonucleoprotein A0, like (*hnrnpa0l*); and extracellular processes such as pyruvate dehydrogenase kinase 2 (*pdk2b*) (Table 2).

IPA analysis of the DEGs following triclosan exposure revealed several pathways of interest. For the 120 h exposure, the top pathways included: xenobiotic metabolism signaling (three genes at both 0.1 and 1 nM); immune system responses, such as NRF2-mediated stress response (two genes at 0.1 and 1 nM, three genes at 1000 nM); metabolic processes, such as cholesterol and glycine synthesis (three genes at 1000 nM); and nervous system organ development and function (five genes at 0.1 nM, two genes at 1 nM) (Table 2). The 24 h triclosan exposure had fewer implicated pathways, but one of the top pathways was organismal injury and abnormalities (33 genes at 0.1 nM, 98 genes at 1 nM, 6 genes at 1000 nM), cancer, specifically the SPINK1 pancreatic cancer pathway, and cardiovascular diseases (16 genes at 1 nM, 1 gene at 1000 nM). Only the lipid metabolism pathway was implicated in both 120 and 24 h triclosan exposure groups. Additionally, there were 10 DEGs expressed in both exposure groups, including: synaptotagmin IV (*syt4*), involved in the nervous system development/signaling pathway; PRELI domain containing 3 (*prelid3b*), involved in the intracellular lipid transport pathway; and perilipin 2 (*plin2*), involved in the lipid metabolism pathway.

### 3.2. Triclocarban

#### 3.2.1. Larval Abnormalities and Mortality

Larval fish exposed to triclocarban for 120 h displayed a significant increase in uninflated swim bladders at the (100 nM) compared to control (*p* < 0.005; Figure 1B). Additionally, total abnormalities were approaching significance (*p* = 0.059), primarily due to the swim bladder abnormalities observed in the 100 nM exposure group. However, fish from the 24 h exposure groups did not experience any significant developmental abnormalities. No significant difference was observed in mortality or the percentage of unhatched eggs at any concentration in either the 120- or 24 h triclocarban exposure.

#### 3.2.2. Behavior

In the 120 h triclocarban exposure, fish exposed to the second-lowest concentration (0.1 nM) showed a decrease in distance moved in the dark (*p* < 0.01), while fish exposed to the highest concentration (100 nM) showed a significant increase in distance moved in the dark (*p* < 0.001) compared to control. The 24 h exposure group exhibited a decrease in distance moved in the dark at the 0.01 nM concentration (*p* < 0.001) (Figure 2B). No significant changes in movement were detected during the light cycle following either the 120- or 24 h exposure.

#### 3.2.3. Gene Expression and Pathway Analysis

Triclocarban exposure resulted in significantly more DEGs compared to the other two chemicals with a total of 2019 DEGs with absolute log2-fold changes ≥0.75 and adjusted *p*-values <0.1. There were 1770 DEGs (724 upregulated and 1056 downregulated, with 10 variably regulated depending on concentration) from the 120 h exposure and 249 DEGs (100 upregulated and 149 downregulated) from the 24 h exposure (Table 1, Table S1). A total of 45 genes were commonly dysregulated following both exposure durations, with two pathways implicated across all exposure concentrations and all exposure durations: cancer and organismal injury and abnormalities, both of which included genes such as tyrosinase-related protein 1b (*tyrp1b*), sequestosome 1 (*sqstm1*), pyruvate dehydrogenase kinase 2 (*pdk2*), and heterogeneous nuclear ribonucleoprotein L (*hnrnpl*). Other affected pathways included endocrine system disorders (expressed following the 1 and 10 nM exposures) and molecular transport and small molecule biochemistry (expressed following the 1 nM exposure).

In addition to the 14 genes commonly dysregulated by triclocarban and triclosan (previously described above), triclocarban and 4-nonylphenol commonly dysregulated 27 genes (Figure 3), with the top affected pathway being lipid metabolism, which was dysregulated following both exposure durations to 0.1 nM 4-nonylphenol, short term exposure to 1000 nM nonylphenol, and long-term exposure to 10 nM triclocarban. The small molecule biochemistry pathway was also dysregulated following both exposure durations to 1 nM triclocarban, long term exposure to 10 nM 4-nonylphenol, and short-term exposure to 0.1 and 1000 nM 4-nonylphenol.

### 3.3. 4-nonylphenol

#### 3.3.1. Larval Abnormalities and Mortality

4-nonylphenol exposure, regardless of concentration, did not affect mortality rate following either the 120- or 24 h exposure duration. While fish exposed to 4-nonylphenol for 120 h had no significant abnormalities (*p* > 0.05), the 24 h exposure fish showed significant deficiency in swim bladder development and total abnormalities at the 1000 nM concentration compared to control (*p* < 0.001) (Figure 1C). Global Chi-Square analysis showed increased cardiac edema (*p* < 0.05) and skeletal abnormalities (*p* < 0.001) in the 24 h exposure group, but pairwise comparisons showed no significant difference between any specific concentration compared to the control group. However, the 1 nM concentration was approaching significance compared to control for uninflated swim bladder (*p* = 0.08). The 1000 nM concentration for the 24 h exposure had the highest occurrence of abnormalities: skeletal (21%), uninflated swim bladder (21%), and yolk sac edema (10%). The percentage of unhatched eggs was increased in the 120 h exposure for the 0.1 and 100 nM concentrations compared to control, although it was not significant (*p* > 0.05).

#### 3.3.2. Behavior

Larval fish exposed to 4-nonylphenol moved significantly less in the dark at 0.1 and 10 nM after both the 120 and 24 h exposures, and also at 100 nM after the 24 h exposure (*p* < 0.001). In the light, decreased movement was observed in the 120 h exposure at 0.1 and 100 nM (*p* < 0.001 and *p* < 0.05, respectively), while increased movement was observed in the 24 h exposure at 1 nM (*p* < 0.001) (Figure 2C).

#### 3.3.3. Gene Expression and Pathway Analysis

4-nonylphenol exposure resulted in 93 DEGs with absolute log2-fold changes ≥ 0.75 and adjusted *p*-values < 0.1 across both exposure durations, with 47 upregulated and 46 downregulated (Table 1). The 120 h exposure resulted in 41 DEGs (33 downregulated and 7 upregulated), with 40 dysregulated at 0.1 nM alone, and 1 gene, zgc:92590 (*zgc:92590*), upregulated at both 0.1 and 1000 nM. The 24 h exposure resulted in 52 DEGs, with 34 upregulated and 4 downregulated at 10 nM alone, and the remaining 14 genes dysregulated at 1000 nM (Table S1). The significant gene expression profiles were distinct for each exposure concentration following the 24 h exposure duration. However, three genes were commonly dysregulated following the 120 and 24 h exposure durations: cytochrome P450, family 3, subfamily A, polypeptide 65 (*cyp3a65*), involved in the xenobiotic signaling pathway; amylase alpha 2A (*amy2a*), involved in the metabolic processes pathway; and pyruvate kinase L/R (*pklr*) involved in cardiac development/metal ion binding pathways.

Overall, 4-nonylphenol exposure resulted in fewer differentially expressed pathways compared to triclocarban or triclosan exposures (Table 2). The main pathways implicated following the 120 h exposure were similar to those affected by triclosan and triclocarban exposure, namely lipid metabolism, small molecule biochemistry functions, organismal injury and abnormalities. Cancer was also implicated in the long-term 4-nonylphenol exposure, specifically the SPINK1 pancreatic cancer pathway, with 4 DEGs following 10 nM exposure, including carboxypeptidase A2 (pancreatic; *cpa2*), carboxypeptidase A1 (pancreatic; *cpa1*), carboxypeptidase B1 (tissue; *cpb1*), and chymotrypsinogen B, tandem duplicate 1 (*ctrb2*). In addition to these, another 4 DEGs in the SPINK1 pathway were present after exposure to 0.1 nM 4-nonylphenol: chymotrypsin-like (*ctrl*), chymotrypsin like elastase 2A (*cela2a*), serine protease 2 (*prss2*) and chymotrypsin like elastase 1 (*cela1*) (Table 2). Embryonic development was also an affected pathway following the 24 h exposure duration, and included genes implicated in cardiovascular development, such as transferrin receptor 1b (*tfr1b*), and neurological development, such as synaptogyrin 3b (*syngr3b*), retinal X-arrestin (*arrb3*), and receptor accessory protein 3b (*reep3b*).

## 4. Discussion

Our results show a wide range of responses to these three EDCs, with notable differences in mortality, morphology, neurobehavior, and gene expression between triclocarban and triclosan, despite their relatively similar functions and chemical structures. Results also varied depending on the timing of exposure, elucidating the importance of examining different windows of developmental exposure. For example, triclocarban led to more significant morphological abnormalities in the 120 h exposure group, whereas none were found with triclosan exposure, but this was likely due to the 100% mortality rate seen at the two highest exposure concentrations (100 and 1000 nM) during both 120 and 24 h exposure periods (Figure 1 and Figure 2). Triclosan-induced mortality generally agrees with existing data, which found a dose-dependent decrease in survival rate with significant reductions in mortality at concentrations < 40 μg/L [27]. However, some studies showed relatively lower or no mortality at concentrations >1000 nM in adult zebrafish [28] and other fish species [29,30,31,32], suggesting that developing organisms are more sensitive to triclosan exposure than adults, and different species have variable sensitivity to this contaminant. Some larval zebrafish studies additionally showed relatively lower mortality at equal or higher concentrations to those investigated in the current study, which could potentially be explained by different exposure methods through variations in water renewal or vehicle [33,34]. Our results are also surprising because a recent study predicted that triclocarban would have more adverse effects than triclosan, with a predicted no effect concentration of 0.0147 μg/L versus 0.1757 μg/L for triclosan [35]. Our two highest concentrations of triclosan (100 and 1000 nM) correspond to 43.4 μg/L and 433.9 μg/L respectively, whereas the highest concentration of triclocarban (10 nM) corresponds to 3.2 μg/L, thus potentially explaining why mortality was increased with triclosan, but not triclocarban exposure in this study. 4-nonylphenol mortality followed along the lines of similar studies, which showed no significant mortality in zebrafish embryos exposed from 4 to 168 hpf to concentrations ranging from 0.1–100 μg/L [36].

A decreased percentage of unhatched eggs was another effect seen at 10 nM triclosan, with only 1% of the eggs unhatched at 10 nM compared to 15% for the control group in the 120 h exposure duration. Zebrafish egg hatching is mainly dependent on one enzyme, zebrafish hatching enzyme 1 (*zhe1*) [37]. Although enzymatic activity and therefore zebrafish hatching rate is mainly dependent on development rate and temperature, studies have shown abnormalities in embryo hatching in response to adverse environmental factors, such as glucocorticoids, salinity, and EDCs [38,39]. These outcomes conflict with previous data on triclosan that indicates triclosan has no effect on the hatching rate of zebrafish [40]. While no genes associated with *zhe1* were dysregulated in this study, the gene *cyp2k18*, which is involved in several processes in the body contributing to homeostasis, including exogenous drug catabolic process, organic acid metabolic process, and xenobiotic metabolic process, as well as heme-binding activity, was significantly upregulated after both 1 and 10 nM exposures in the 120 h triclosan exposure group, with an absolute log-fold change of 2.4 in the 10 nM group. Although no literature specifically links this gene to a decreased percentage of unhatched eggs, *cyp2k18* upregulation is considered a marker of toxicity and stress. For example, *cyp2k18* is significantly upregulated due to drug toxicity [41], and *cyp2k18* transgenic zebrafish have been developed in order to assess toxicity to chemotherapy drugs [42]. Finally, upregulation of *cyp* genes has been linked to tumorigenesis in both murine and Japanese medaka (*Oryzias latipes*) models [43]. Therefore, upregulation of *cyp2k18* is likely a marker of triclosan toxicity in the current study and should be explored further as a potential marker of adverse environmental factors.

Triclocarban and 4-nonylphenol both showed a significant increase in uninflated swim bladders at the highest exposure concentration (100 nM), with total abnormalities for triclocarban approaching significance mainly due to the swim bladder abnormalities observed in the 100 nM exposure group. For 4-nonylphenol, there was a trend of swim bladder abnormalities in all concentrations of the 24 h exposure, but only the 1000 nM concentration was significant. Several DEGs expressed across all concentrations in the long term triclocarban exposure group included *sqstm1*, involved in axogenesis and nervous system development, and *pdk2*, which is involved in glucose metabolism in the mitochondria. Both genes are involved in the organismal injury and abnormalities pathway. Although no studies link *sqstm1* upregulation specifically to swim bladder deflation, upregulation has been linked to tumorigenesis in bronchial epithelial cells in humans [44], while *pdk2* upregulation has been linked to the development of pulmonary hypertension [45]. The zebrafish swim bladder shares many developmental [46] and transcriptomic [47] traits with the human pulmonary system, so upregulation of these genes could be contributing to the deflated swim bladders and overall abnormalities seen with the long-term triclocarban exposure. Abnormalities in blood circulation and oxygen delivery have been implicated in uninflated swim bladders in zebrafish [48], and downregulation of *pklr*, which is expressed in red blood cells, occurred at short term 1000 nM 4-nonylphenol exposure.

4-nonylphenol-induced abnormalities were only seen following the 24 h exposure, with the most DEGs following the 24 h exposure at 10 and 1000 nM concentrations (41 genes), compared to the 120 h exposure period (2 genes). This illustrates that the specific window of susceptibility is particularly important 4-nonylphenol toxicity. Cardiac edema was another abnormality noted after short term 4-nonylphenol exposure; however, it was only significant with global Chi-Square analysis. When pairwise comparison was conducted, no significant difference in cardiac edema between individual concentration levels and the control group occurred. 4-nonylphenol exposure following the 24 h exposure period resulted in the most DEGs related to the cardiovascular system, such as *tfr1b*, which is responsible for hemoglobin biosynthesis, significantly upregulated at 10 nM and approaching significance at 0.1 nM. Resulting hemoglobin defects can lead to increased viscosity and cardiac edema, as seen in a study exposing African catfish (*Clarias gariepinus*) to concentrations of 4-nonylphenol ranging from 250 to 1000 μg/L [49].

Behavioral abnormalities resulted from exposure to each of the three chemicals, with triclosan exposure resulting in the most behavioral changes, followed by 4-nonylphenol, and then triclocarban. Zebrafish are more active in the dark [50] as they search for better lit environments where they can better identify food sources and increase their likelihood of survival [51]. Our triclosan behavioral results for the 120 h exposure are similar to previous findings of hypoactivity in response to triclosan exposure in a variety of aquatic organisms, mice, and humans [40,52,53,54,55,56,57]. We have expanded upon this previous research by demonstrating that this hypoactivity response is present at lower concentrations (0.1 nM, 1 nM, and 10 nM; Figure 2A) than previously tested (10 μg/L to 0.6 mg/L). This hypoactivity may be related to neurological dysfunction associated with dysregulation of *syt4*, which binds phospholipids in the nervous system, and *prelid3b*, which transports lipids in the nervous and musculature systems, which are both differentially expressed following the long- and short-term triclosan exposures. Conversely, the 24 h triclosan exposure resulted in hyperactivity at only the 1 nM concentration during the dark cycle and at 10 nM during the light cycle. Two existing studies also found hyperactivity in response to triclosan exposure in zebrafish [58] and humans (boys, but not girls) [59]. In both studies, exposure to higher concentrations of triclosan, or a mixture of triclosan and its metabolites, seemed to precipitate the hyperactive behavior, though both exposure periods were much longer than the exposure period in the current study. The need for further research into how triclosan exposure duration and concentration affects behavior is highlighted by the differential effects seen within our study, namely the lower concentrations inducing hypoactive behavior following embryonic exposure, but hyperactivity following larval exposure.

Hypoactivity was the main behavioral change seen in response to 4-nonylphenol in both the 24 and 120 h exposures. 0.1 and 10 nM showed hypoactivity from both exposure time periods, but in the 24 h exposure, the most significant hypoactivity was at the highest concentration (1000 nM) during the dark cycle. Activity in the light phases was generally decreased as well, though only significantly for long term exposure at the 0.1 and 100 nM concentrations. These results agree with previous 4-nonylphenol studies showing some level of hypoactivity in response to exposure in mice and fish [60,61,62,63,64,65]. In contrast, only the 21 nM exposure resulted in hyperactivity during the light phase, although it is unclear why this occurred.

A non-monotonic response was seen in triclocarban behavior from the 24 h exposure, which only resulted in hypoactivity at 0.01 nM. Conversely, in the 120 h exposure in the dark phase, the second lowest concentration resulted in hypoactivity while the highest concentration resulted in hyperactivity. There have not been many studies on the effects of triclocarban exposure on behavior. One study of fathead minnows (*Pimephales promelas*) found reduced aggression in adult males at 560 and 1576 ng/L and no change in larval behavior [66], while a study of *Gammarus locusta* found reduced activity in females at 500 ng/L [67].

Finally, IPA analysis revealed many pathways common to all chemicals and a few unique to each chemical. The top pathways shared by all chemicals involved metabolic processes (Table 2), such as lipid metabolism, proteolysis, and cholesterol and glycine synthesis. Out of the 49 differentially regulated genes shared in various combinations between triclosan, triclocarban, and 4-nonylphenol, the majority were involved in metabolic processes, specifically with the pancreas, such as *cel.1*, implicated in lipomatosis and diabetes, *amy2a* involved in carbohydrate metabolism, *prss59.2* implicated in pancreatitis, *el2a* involved in proteolysis, and *zgc:92590* involved in proteolysis. The upregulation of these genes is mainly seen in triclosan and 4-nonylphenol, which may be contributing to the pancreatic cancer pathway seen with these chemical exposures. The SPINK1 pancreatic cancer pathway is thought to increase risk of pancreatic cancer by lowering the activation threshold of trypsin [68]. SPINK1 is believed to promote proliferation of cancer cells by inducing EGFR phosphorylation, which results in activation of the mitogen-activated protein kinase (MAPK) pathway [69]. Additionally, the SPINK1 pathway activates the NRF2 pathway, which leads to increased proliferation and decreased apoptosis of cancer cells [70]. Although the SPINK1 gene itself was not dysregulated within our study, genes related to the SPINK1 and the NRF2 pathway, namely abcc2, were dysregulated. The gene *abcc2*, which is implicated in multiple forms of carcinomas, was upregulated in both triclosan and triclocarban exposures, specifically the long-term exposures for triclosan 10 nM and triclocarban 100 nM. Triclosan has been implicated as inducing metabolic acidosis and regressing pancreatic islet cells into pycnotic cells leading to cell death [71], as well as various cancers such as liver and breast tumors [72]. Additionally, chymotrypsin C in humans has also been implicated in pancreatic cancer [73], and the orthologue in zebrafish, *ela2l* was upregulated in the short-term duration exposure for both 4-nonylphenol at 10 nM and triclosan at 10 nM. Endocrine disruptors are commonly known to contribute to endocrine disruption leading to disease processes such as obesity, type 2 diabetes and metabolic syndromes [74], so it is unsurprising that the metabolic pathways listed above were shared by all three chemicals examined within this study.

In conclusion, the findings of the present study expand upon and contribute to the limited studies of developmental-toxicity regarding exposure to 4-nonylphenol, triclosan and triclocarban in embryogenesis and larval zebrafish. Furthermore, our data indicates several future areas of exploration, such as the metabolic effects of chemical exposure, including potential pathways leading to pancreatic cancer, and disorders such as diabetes and hepatitis, as well as the potential impacts of gene differentiation on cardiovascular abnormalities and behavior. Our results indicate that environmentally relevant levels of exposure can disrupt neurologic, behavioral, cardiovascular and metabolic pathways, potentially leading to adverse health outcomes such as cardiac edema and significant mortality and should be explored further. Overall, our results indicate the potential for gene expression changes and population impacts, depending on the time of exposure, caused by these three known endocrine disruptors, and these potential impacts should be studied further not only in aquatic lifeforms, but human health as well.

## Figures and Tables

**Table 1:** Number of significantly dysregulated genes (significance defined as absolute log2-fold changes ≥ 0.75 and adjusted *p*-value < 0.1) in zebrafish following extended duration 4NP, TCC or TCS exposure starting at 4 h post-fertilization through 5 days post-fertilization, short term duration starting at 4 dpf through 5 dpf, and total genes dysregulated across all concentrations for extended duration and short term duration. ↓ indicates gene downregulation; ↑ indicates gene upregulation.

		Triclosan		
	0.1 nM	1 nM	10 nM	Total genes
24 h	7 ↓, 15 ↑	2 ↓, 17 ↑	1 ↓, 3 ↑	10↓ 35↑
120 h	11 ↓, 8 ↑	0 ↓, 1 ↑	3 ↓, 8 ↑	14 ↓ 17↑
		**Triclocarban**		
	0.01 nM	1 nM	100 nM	Total genes
24 h	115 ↓, 54 ↑	26 ↓,37 ↑	8 ↓, 9 ↑	149↓ 100↑
120 h	0 ↓, 1 ↑	578 ↓, 258 ↑	478 ↓, 465 ↑	1056↓ 724↑
		**4-nonylphenol**		
	0.01 nM	10 nM	1000 nM	Total genes
24 h	0 ↓, 0 ↑	4 ↓, 34 ↑	8↓, 6 ↑	12 ↓ 40↑
120 h	32 ↓, 7 ↑	1↓, 0 ↑	1 ↓, 0 ↑	33 ↓ 7↑

**Table 2:** Differential expression for all genes altered in zebrafish following extended duration 4NP, TCC or TCS exposure starting at 4 h post-fertilization through 5 days post-fertilization and short-term duration starting at 4 dpf through 5 dpf. Significant absolute log2-fold changes (value ≥ 0.75 and adjusted *p*-value < 0.1) in bold. (ND = no difference in expression). Purple background indicates significant differential expression across all 3 chemicals, yellow across 4NP and TCS, green across 4NP and TCC, and blue across TCC and TCS.

		Exposure Concentrations (nM) 0–5 Days									Exposure Concentrations (nM) 4–5 Days								
	Gene Name	4-nonylphenol			Triclosan			Triclocarban			4-nonylphenol			Triclosan			Triclocarban		
		0.1	10	1000	0.1	1	10	0.01	1	100	0.1	10	1000	0.1	1	10	0.01	1	100
Cardiovascular																			
*fbxo32*	F-box protein 32	**−1.2**	−0.8	−0.2	0.4	0.2	1	−0.01	0.5	**1.4**	0.6	0.2	−0.5	**1.1**	0.3	0.1	**1.7**	−1.1	−0.2
*tfr1b*	transferrin receptor 1b	0.4	0.2	0.3	0.1	−0.1	0.2	−0.03	−0.1	**−1.2**	0.6	**0.8**	−0.2	0.2	0.1		−0.4	−0.9	
*myl10*	myosin, light chain 10, regulatory	−0.1	0.04	−0.3	−0	0.1	−0	−0.2	0.2	−0.1	0.3	0.4	**0.8**	0.2	0.2	−0.1	**−0.8**	−0.7	0.1
*mat2aa*	methionine adenosyltransferase II, alpha a	−0.1	0.1	0.02	−0	−0	0.4	−0.01	−0.5	**−1**	−0.1	0.2	**0.9**	−0.1	0.2	0.3	−0.1	0.2	0.05
hbae1.1	hemoglobin, alpha embryonic 1.1	−0.1	0.01	−0.3	−0.8	−0.3	−0.3	0.1	−0.6	−0.4	−0.4	−0.2	**0.9**	−0.6	**1.1**	**1**	**−0.7**	−1.6	−0.2
*tcp1*	t-complex 1	0.3	−0.2	−0.1	**−0.9**	−0.6	−0.2	0.2	**−1.2**	−0.2	−0.1	−0.3	0.2	**−1**	0.3	0.3	−0.3	−0.1	−0.2
*hbbe1.2*	hemoglobin beta embryonic- 1.2	0.2	0.3	0.04	ND	−0.3	−0.4	0.04	−0.1	−0.3	−0	−0.2	0.03	−0.1	**1.1**	0.4	−0.3	−0.8	**−0.8**
Neurological																			
*reep3b*	receptor accessory protein 3b	0.5	0.7	0.2	0.6	0.03	0.2	−0.4	0.2	−0.2	0.5	**0.8**	0.2	**0.8**	0.1	0.01	−0.3	−1.4	0.1
*syngr3b*	synaptogyrin 3b	−0.03	0.7	0.3	**0.9**	0.1	0.2	−0.1	0.3	−0.3	0.5	**1**	0.1	**0.9**	0.1	−0.1	0.3	−0.2	−0.04
*agr2*	anterior gradient 2	**0.9**	0.4	−0.2	−0.5	−0.4	−0.3	0.2	−0.7	**−0.8**	−0.1	−0.05	0.4	−0.3	0.4	0.05	−0.7	−2.5	−0.1
*crygm2d18*	crystallin, gamma M2d18	−0.2	**−1**	−0.1	0.2	0.1	−0.8	0.03	0.04	−0.1	−0.3	0.3	−0.1	−0.6	0.6	0.1	−0.2	−1	**0.8**
*elmo2*	engulfment and cell motility 2	−0.2	0.4	−0.1	0.2	−0	0	−0.3	−0	−0.2	0.4	**0.8**	−0.04	0.4	0.5	−0.1	0.5	1.4	0.02
*arr3b*	arrestin 3b, retinal (X- arrestin)	0.1	0.4	−0.02	−0.1	0.2	0.1	−0.3	−0	**−2.6**	0.2	**0.8**	−0.2	0.1	−0.1	−0.2	0.04	−0.4	0.2
*opn1sw2*	opsin 1 (cone pigments), short-wave-sensitive 2	−0.1	−0.01	0.2	−0.2	−0.3	**−0.8**	−0.1	−0.1	−0.3	−0.3	−0.4	0.2	−0.4	−0.4	−0.3	**−0.8**	**−1.3**	−0.1
*si:ch211- 153b23.5*	si:ch211-153b23.5	0.9	0.3	−0.2	**−1**	−0.9	−0.6	0.1	**−0.9**	0.6	0.7	0.5	1	−0.8	0.8	0.1	−0.4	0.4	0.7
*mgst3a*	microsomal glutathione S- transferase 3a	0.3	−0.1	0.1	**−0.9**	−0.9	−0.3	0.2	**−1.1**	−0.5	−0.5	−0.5	−0.1	**−1**	0.4	0.3	0.02	−0.5	−0.4
*rlbp1b*	retinaldehyde binding protein 1b	0.1	0.1	0.4	−0	−0	0.2	0.2	0.1	**−0.8**	0.2	−0.1	0.1	0.4	**1.5**	0.5	**−1.1**	−0.5	0.04
Metabolic Processes																			
*cel.1*	carboxyl ester lipase, tandem duplicate 1	**−1.2**	−0.7	−0.3	0.6	0.4	0.2	−0.3	0.5	0.4	0.1	−0.3	−0.5	**1**	0.2	−0.3	0.05	−0.6	−0.5
*amy2a*	amylase alpha 2A	**−0.9**	−0.7	−0.1	**0.8**	0.4	0.2	−0.1	−0.2	0.5	0.1	−0.2	**−0.8**	0.8	0.4	−0.2	0.4	0.4	−0.6
*prss59.2*	serine protease 59, tandem duplicate 2	**−0.8**	−0.4	0.04	0.3	−0.1	0.2	−0.1	**−1**	0.1	−0	−0.2	−0.4	0.4	**1.1**	0.2	0.1	−0.3	−0.4
*ela2l*	elastase 2 like	−0.7	−0.3	0.06	0.3	−0.5	−0.6	0.2	−0.2	−0.1	−0.5	**−0.9**	−0.4	0.5	0.4	**−0.9**	0.1	−0.8	−0.3
*pip5k1cb*	phosphatidylinositol-4- phosphate 5-kinase, type I, gamma b	0.4	0.7	0.4	0.6	0.3	0	−0.4	0	−0.2	0.5	**0.9**	0.1	**0.8**	−0.2	−0.3	0.7	−0.3	0.4
*fabp10a*	fatty acid binding protein 10a, liver basic	**−0.9**	−0.1	−0.1	0.2	0.01	0.3	−0.1	0.04	−0.1	−0.1	−0.02	−0.7	0.4	0.6	0.2	0.4	−0.9	−0.2
*zgc:92590*	*zgc:92590*	**−0.9**	−0.3	**−1.1**	−0	−0	0	−0.1	−0.3	**−1.6**	−0.1	0.2	−0.2	0.1	0.8	0.1	−0.03	−1.1	**−0.9**
*si:ch211- 234p6.10*	si:ch211-234p6.10	**−0.8**	−0.03	0.4	0	−0.2	0.2	−0.04	0.1	−0.3	0.4	0.1	**−0.6**	0.3	0.6	0.2	**0.9**	0.3	−0.4
*lpin1a*	lipin 1	**−0.8**	−0.4	−0.4	0.1	−0	0.2	−0.2	0.2	**0.9**	0.2	−0	−0.3	0.4	0.02	−0.1	0.6	0.2	−0.03
*fkbp9*	FKBP prolyl isomerase 9	0.8	0.6	−0.02	−0.1	−0.3	0	−0.4	**−1.7**	**−2**	−0	**1**	0.8	−0.2	−0.2	0.04	−0.1	−0.6	−0.1
*abcc2*	ATP-binding cassette, sub- family C (CFTR/MRP), member 2	−0.4	0.1	−0.1	0.4	1	**1**	−0.1	0.4	**1**	0.3	0.2	0.2	0.7	−0.2	0.6	−0.1	0.2	−0.1
*plin2*	perilipin 2	0.06	−0.2	0.6	−0	0.1	**1.1**	−0.2	−0.3	**1.1**	0.8	0.4	0.4	0.3	1	**1.1**	0.4	−0.6	−0.1
*fdps*	farnesyl diphosphate synthase (farnesyl pyrophosphate synthetase, dimethylallyltranstransferas e, geranyltranstransferase)	0.1	0.4	0.1	−0.1	−0.4	0.4	0.02	−0.1	**−1.2**	0.03	0.4	0.3	0.5	**1**	0.6	0.04	0.3	0.1
Immune System																			
*irg1l*	immunoresponsive gene 1, like	**1.5**	0.2	0.04	−0.5	−0.2	−0.2	0.2	**−0.7**	**1.8**	0.8	0.4	0.6	−0.3	0.8	0.2	−0.3	0.6	1.1
*hsp90b1*	heat shock protein 90, beta (grp94), member 1	**1**	0.2	0.3	−0.3	−0.1	−0.2	0.4	**−0.9**	0.2	0.1	−0.1	0.4	−0.4	−0.4	0.2	−0.3	−0.9	−0.1
*ctsl.1*	cathepsin L.1	−0.4	0.2	−0.4	0.7	0.3	−0.1	−0.1	−0.2	0.7	0.6	0.5	**−1.3**	0.8	0.5	−0.5	**1**	−0.1	−0.4
Extracellular Processes																			
*si:dkey-14d8.6*	si:dkey-14d8.6	**−1.1**	−0.4	0.01	**1**	0.4	0	−0.2	−0.2	0.5	0.2	−0.2	−0.3	0.9	0.4	−0.5	0.5	−0.2	−0.4
*pdk2b*	pyruvate dehydrogenase kinase 2b	**−1.1**	−0.5	0.004	−0	0.2	**0.9**	−0.1	0.3	**1.2**	0.5	−0.2	−0.5	0.3	0.4	0.3	**0.8**	0.5	−0.6
*cpa1*	carboxypeptidase A1 (pancreatic)	**−1.3**	−0.9	−0.5	0.1	0.02	0.3	−0.1	−0.6	−0.2	−0.1	−0.3	−0.6	0.3	0.4	0.3	**0.9**	−0.8	−0.4
Intracellular Processes																			
*si:ch211- 153b23.4*	si:ch211-153b23.4	**1.2**	−0.2	−0.3	−0.9	−0.5	−0.5	0.2	−0.4	**1.4**	0.8	0.1	0.8	−0.5	**1.2**	0.5	−0.3	0.05	0.5
*mt-tRNA*	tRNA on mitochondrial genome	−0.002	−0.04	−0.05	**−0.9**	−0.4	−0.6	0.1	0.4	0.5	−0.3	**−1.1**	−0.4	−0.4	0.2	0.1	**−1**	−1.9	−0.4
*prelid3b*	PRELI domain containing 3	0.1	0.4	0.3	**1**	0.4	0.5	−0.4	0.1	−0.5	0.4	0.8	0.1	**1.3**	0.3	−0.1	0.2	−0.6	0.2
*hnrnpa0l*	heterogeneous nuclear ribonucleoprotein A0, like	0.4	0.2	−0.03	**−0.8**	−0.5	−0.3	0.001	**−1**	**−0.8**	0.2	0.5	**1**	**−0.8**	0.5	0.1	−0.1	0.1	−0.02
*calcoco1b*	calcium binding and coiled- coil domain 1b	**−1**	−0.7	−0.3	0.1	0.1	0.4	0.1	0.7	**2.2**	0.4	−0.2	−1.3	0.7	0.7	0.2	**0.9**	1.1	−0.1
*mknk2b*	MAPK interacting serine/threonine kinase 2b	**−0.9**	−0.5	−0.2	0	0.1	0.2	0.03	−0.1	**1.1**	−0	−0.2	−0.4	0.3	0.1	−0	0.6	−0.1	0.1
*trim63a*	tripartite motif containing 63a	**−0.8**	−0.6	−0.2	−0.3	−0.1	0.1	0.2	0.3	**0.9**	0.04	−0.3	−0.6	0.1	0.2	−0.2	0.5	−1.6	−0.2
*si:ch211- 207n23.2*	si:ch211-207n23.2	**0.9**	0.3	0.03	−0.5	−0.4	−0.5	0.3	−0.3	**1.5**	0.8	0.5	0.2	−0.3	0.5	0.1	0.2	0.3	0.4
*si:ch211- 153b23.3*	si:ch211-153b23.3	**1.2**	−0.02	−0.1	−0.1	−0	−0	0.2	0.3	**1.9**	0.3	0.3	0.5	−0	0.7	0.3	−0.2	0.8	**1.3**
*sult5a1*	sulfotransferase family 5A, member 1	**1.7**	0.7	0.1	−0.1	−0	−0.1	0.1	−0.2	**2.2**	0.4	0.3	0.2	−0.1	1	0.1	−0.3	0.9	0.7
*mt-atp8*	ATP synthase 8, mitochondrial	−0.5	−0.4	−0.7	−0.4	0.2	0	−0.2	**0.8**	0.1	−0.7	**−1**	0.2	−0.3	−0.4	0	−0.5	0.2	−0.1
*dgcr8*	DGCR8 microprocessor complex subunit	0.1	0.4	0.02	0.2	0.2	0.3	−0.2	0.3	**−0.8**	0.3	**0.8**	0.1	0.1	−0.02	0.1	0.1	0.6	0.1
*bub3*	BUB3 mitotic checkpoint protein	0.2	0.3	−0.04	−0.2	−0.1	−0.1	−0.1	**−1.3**	**−1.1**	0.3	**0.8**	0.7	−0.1	−0.1	−0.1	−0.3	−1	0.5
*xpot*	exportin, tRNA (nuclear export receptor for tRNAs)	−0.3	0.2	−0.3	0.1	−0	−0.1	−0.4	**−0.8**	**−1.5**	0.3	**0.8**	0.4	−0	−0.1	−0.2	0	0.4	**0.9**
*si:ch211- 250g4.3*	si:ch211-250g4.3	0.1	0.3	0.3	0.7	0.4	0.7	0.1	**0.8**	**1**	0.3	**0.8**	−0.05	0.8	0.5	0.3	**0.8**	−0.2	−0.02
*cebpd*	CCAAT enhancer binding protein delta	−0.1	0.03	0.04	−0.4	0.1	0.6	0.2	−0.2	**1.2**	0.1	−0.5	**−0.8**	0	0.4	0.4	**0.9**	0.01	−0.2
*hnrnpa0a*	heterogeneous nuclear ribonucleoprotein A0a	0.5	0.2	−0.1	−0.5	−0.4	−0.4	−0.1	−0.5	**−0.8**	0.2	0.4	**0.8**	−0.8	0	0.02	−0.02	−0.4	−0.3
*fkbp5*	FKBP prolyl isomerase 5	−0.4	0.1	0.1	0.4	0.7	**1**	0.2	−0.1	0.2	0.01	−0.5	0.5	0.5	−0.4	ND	**0.8**	−0.02	−0.03
*ddx39ab*	DEAD (Asp-Glu-Ala-Asp) box polypeptide 39Ab	0.3	−0.1	−0.1	**−1**	−0.6	−0.4	0.1	−0.6	**−0.9**	−0.1	−0.2	0.3	**−1.2**	0.3	0.3	−0.6	−0.9	−0.2
*tyrp1b*	tyrosinase-related protein 1b	0.4	−0.02	0.02	**−1**	−0.8	−0.4	0.2	−0.2	**−1.5**	−0.3	−0.4	−0.1	−0.7	0.4	0.2	**−1**	−0.4	−0.2
*hnrnpa0b*	heterogeneous nuclear ribonucleoprotein A0b	0.5	0.2	0.3	**−0.8**	−0.5	0.2	0.2	**−0.9**	**−1.3**	−0.2	−0.01	0.2	−0.6	0.3	0.4	0.1	0.6	−0.1
*phtf2*	putative homeodomain transcription factor 2	−0.4	0.2	0.2	0.5	0.2	0.2	−0.2	0.3	−0	0.3	0.3	−0.1	**0.9**	0.2	0	−0.2	−2.4	0.2
*zmp:0000001081*	zmp:0000001081	0.2	0.1	0.3	0.7	0.3	0.6	0.2	0.2	**1.4**	0.2	0.7	−0.3	**0.9**	0.5	0.2	**0.8**	0.2	0.2
*si:ch211- 113a14.18*	si:ch211-113a14.18	−0.003	−0.1	0.02	0.1	0.3	0.1	0.1	0.7	0.1	−0.1	−0.2	−0.3	−0.1	**−1.4**	−0.5	0.2	**2.4**	−0.1
*zgc:113263*	zgc:113263	−0.1	−0.2	0.1	0.2	0.01	0	−0.1	0.2	0.4	0.3	0.2	0.2	0.01	**−1.2**	−0.2	0.2	1.4	0.1
*si:ch211- 132b12.7*	si:ch211-132b12.7	0.1	0.4	−0.1	0.2	0	0.7	−0.3	−0.2	**−2**	−0.2	0.5	−0.1	0.9	**1**	0.5	0.1	−0.03	−0.1
*tm4sf21b*	transmembrane 4 L six family member 21b	−0.02	−0.4	−0.2	−0.1	−0	0.2	0.3	**−1**	−0.4	−0.5	−0.5	0.1	0.1	**1.3**	0.6	−0.3	0.2	−0.6
Xenobiotic Signaling																			
*cyp3a65*	cytochrome P450, family 3,subfamily A, polypeptide 65	**−1**	−0.6	−0.1	0.5	**1**	0.7	0.1	0.6	**2**	0	−0.5	**−0.8**	0.5	0.1	0.1	−0.1	−0.6	−0.4
*ucp1*	uncoupling protein 1	**−0.9**	−0.4	0.1	0.5	0.1	0.4	−0.1	0.3	**1.4**	0.1	−0.2	−0.5	0.7	0.9	0.1	0.7	−0.1	−0.5
*cyp2k18*	cytochrome P450, family 2, subfamily K, polypeptide18	0.3	−0.03	0.2	0.2	**2**	**2.4**	−0.1	−0.1	**1.9**	0.2	−0.2	0.6	0.1	−0.2	0.3	−0.2	2.4	−0.5

## Data Availability

The data presented in this article are available within the text and Supplementary Materials.

## Supplementary Materials

The following are available online at https://www.mdpi.com/article/10.3390/toxics10020053/s1, Table S1: Significant gene expression changes (Log2 fold change and *p*-value) results of zebrafish embryos following exposure to triclosan, triclocarban or 4 nonylphenol.
